# Supplementary material for: Application of a 72 h National Early Warning Score and Incorporation with Sequential Organ Failure Assessment for Predicting Sepsis Outcomes and Risk Stratification in an Intensive Care Unit: A Derivation and Validation Cohort Study
Source: J Pers Med. 2021 Sep 13;11(9):910. doi: 10.3390/jpm11090910 (PMC8465191; doi:10.3390/jpm11090910)
Supplement: Supplementary file 1 [file jpm-11-00910-s001.zip › jpm-1324477-supplementary.pdf]

Supplement

Table. S1 Comparison of discrimination for mortality at different times

AUC/cut-off point	APACHEII	Adm-qSOFA	Adm-SOFA	Adm-NEWS2	*p*-Value
Mortality in Day 7	0.578 / >25	0.537 / >1	0.629* / >10	0.566 / >8	0.014[a]
Mortality in Day 14	0.552 / >21	0.520 / >2	0.625* / >11	0.568*/ >8	0.012[a]
Mortality in Day 21	0.569*/ >24	0.528 / >2	0.600* / >11	0.581*/ >11	0.042[a]
Mortality in Day 28	0.566*/ >24	0.525 / >1	0.593* / >12	0.569*/ >11	0.040[a]

*significance level

[a]significant difference was only shown between SOFA and qSOFA

AUC: area under the curve, Adm: admission, APACHEII: Acute Physiological Assessment and Chronic Health Evaluation II, SOFA: Sequential Organ Failure Assessment, NEWS2: National Early Warning Score 2

Table. S2 Comparison of discrimination from NEWS2 and SOFA at admission and on day 3

AUC/cut-off point	Adm-NEWS2		Day-3 NEWS2		Adm[1]-SOFA		Day-3 SOFA
Mortality in Day 7	0.566* / >8		0.741* />8		0.629* / >10		0.740* />11
Pairwise comparison[2]		p < 0.001		p =0.03		p < 0.001	-
Mortality in Day 14	0.568* / >8		0.657* />6		0.625* / >11		0.680* />10
Pairwise comparison[2]		p < 0.001		p =0.409		p = 0.029	
Mortality in Day 21	0.581* / >11		0.669* />6		0.600* / >11		0.684* />8
Pairwise comparison[2]		p =0.005		p =0.052		p < 0.001	
Mortality in Day 28	0.569* / >11		0.649* />6		0.593* / >12		0.677* />8
Pairwise comparison[2]		p < 0.001		p =0.094		p < 0.001	

*significance level

AUC: area under the curve, Adm: admission, APACHEII: Acute Physiological Assessment and Chronic Health Evaluation II, SOFA: Sequential Organ Failure Assessment, NEWS2: National Early Warning Score 2

Table S3. Mortality at different endpoints in derivation cohort

	Low-risk	Intermediate-risk	High-risk	*p*-Value
Mortality within 7 days	1.6%	5.8%	26.4%	<0.001*
Mortality within 14 days	5.6%	15.4%	42.5%	<0.001*
Mortality within 21days	8.4%	22.9%	49.4%	<0.001*
Mortality within 28 days	12.2%	26.0%	56.3%	<0.001*

*Significant difference among groups

Table S4. Mortality at different endpoints in validation cohort

	Low-risk	Intermediate-risk	High-risk	*p*-Value
Mortality within 7 days	1.4%	5.8%	21.9%	<0.001*
Mortality within 14 days	4.1%	14.7%	32.8%	<0.001*
Mortality within 21days	8.1%	21.6%	43.8%	<0.001*
Mortality within 28 days	11.0%	38.4%	54.7%	<0.001*

*Significant difference among groups

Table S5. Ventilator-days and ICU-days for different risk stratification

	Low-risk	Intermediate-risk	High-risk	*p*-Value
ICU-days	7.0(4.8-12.4)	11.7(6.5-17.9)	14.0(6.6-23.3)	<0.001
Ventilator-days	9.1(5.0-22.0)	15.0(7.8-30.9)	20.5(7.3-33.7)	<0.001

All values as median (IQR). ICU: intensive care unit.
